# Gender differences in major depressive disorders: A resting state fMRI study

**DOI:** 10.3389/fpsyt.2022.1025531

**Published:** 2022-11-10

**Authors:** Zhaoyuan Tu, Feng Wu, Xiaowei Jiang, Lingtao Kong, Yanqing Tang

**Affiliations:** ^1^Department of Psychiatry, The First Hospital of China Medical University, Shenyang, China; ^2^Department of Radiology, The First Hospital of China Medical University, Shenyang, China; ^3^Brain Function Research Section, The First Hospital of China Medical University, Shenyang, China; ^4^Department of Gerontology, The First Hospital of China Medical University, Shenyang, China

**Keywords:** ReHo, gender, magnetic resonance imaging, occipital lobe, ALFF, dorsolateral superior frontal gyrus, median cingulate gyrus

## Abstract

**Background:**

Major depressive disorder (MDD) has a high disability rate and brings a large disease burden to patients and the country. Significant sex differences exist in both the epidemiological and clinical features in MDD. The effect of sex on brain function in MDD is not clear now. Regional homogeneity (ReHo) and ALFF are widely used research method in the study of brain function. This research aimed to use ReHo and ALFF to explore gender differences in brain function images in MDD.

**Methods:**

Eighty first-episode drug-naive patients (47 women and 30 men) with MDD and 85 age, education matched healthy volunteers (47 women and 31 men) were recruited in our study and participated in resting-state functional magnetic resonance imaging scans. ReHo and ALFF were used to assess brain activity, two-way ANOVA and *post hoc* analysis was conducted to explore the sex difference in MDD. Correlation analysis was used to explore the relationship between abnormal brain functioning and clinical symptoms.

**Results:**

We observed sex-specific patterns and diagnostic differences in MDD Patients, further *post hoc* comparisons indicated that women with MDD showed decreased ALFF value in the right superior occipital gyrus and decreased ReHo value in the left calcarine and left dorsolateral superior frontal gyrus compared with HC females and men with MDD. Men with MDD showed decreased ReHo value in the right median cingulate gyrus compared with HC males and increased ReHo value in the left dorsolateral superior frontal gyrus compared with HC males, we also found that HC males showed higher ReHo value in the right median cingulate gyrus than HC females.

**Conclusions:**

Men and women do have sex differences in brain function, the occipital lobe, calcarine, DLPFC, and DCG were the main different brain regions found between male and female in MDD, which may be the biomarker brain regions that can help diagnose and treat MDD in men and women.

## Introduction

Major depressive disorder (MDD) is a multifactorial disease of unknown etiology involving genetics, endocrine factors, chronic inflammation and other aspects. It is mainly characterized by a loss of interest, anhedonia, and decreased energy and may be accompanied by sleep disorders, eating disorders, self-injury, and suicidal thoughts and behaviors (1). According to the World Health Organization (WHO), depression accounts for 10% of the world's total non-fatal disease burden (2). A Chinese epidemiological study found that the incidence of major depression in various regions of China has increased each year, with a lifetime prevalence rate of 3.9% (3). Epidemiological data suggest a significant sex difference in the prevalence of MDD (4–6). A previous meta-analysis found that the incidence of MDD in women was approximately twice as high as that in men (7), but the reason for this difference is currently unclear. Nevertheless, genetic factors, sex hormones, and heightened exposure to severe adversity may be responsible for this difference (8). The clinical manifestations of depression also differ by sex. Women with MDD are more likely to report anxiety symptoms, guilt, atypical depression and somatic symptoms (9, 10), whereas men are more likely to experience suicidal and addictive behaviors (11). These differences in clinical manifestations affect the correct diagnosis and treatment of MDD in different sexes. Therefore, exploring the sex differences in MDD and finding objective indicator differences can contribute to further identification and treatment of MDD.

Previous studies (12, 13) have found that MDD may lead to biological changes, including neurotransmitter imbalance, impaired neurogenesis, decreased neuroplasticity, and abnormal neural circuits. These abnormalities suggest a possible physiological indicator of MDD. The development of magnetic resonance technology has allowed magnetic resonance to become an important imaging method to explore neurological diseases with several advantages, such as non-invasiveness, high sensitivity, and high resolution, and has been widely used in the study of various psychiatric disorders such as obsessive-compulsive disorder (OCD) (14, 15), schizophrenia (16), bipolar disorder (17), depression (18, 19) and so on. Relevant studies have shown that various clinical manifestations of MDD are closely related to changes in structural brain imaging and functional brain imaging (13). Specifically, the cortico-limbic-striatal neural system is the main abnormal brain region in patients with MDD (12, 20). Furthermore, sex differences in the cortico-limbic-striatal neural system have been shown to contribute to sex differences in psychiatric disorders. Functional magnetic resonance imaging (FMRI) is an important method for brain functional imaging research. Blood oxygenation level dependence (BOLD) is a commonly used imaging method for FMRI (21). BOLD mainly uses deoxyhemoglobin (deoxyhemoglobin) as a comparison to take signals of cerebral blood oxygen and cerebral blood flow. Different activities of neurons in the brain can cause changes in cerebral oxygen uptake as well as corresponding changes in blood flow and the conversion of deoxyhemoglobin and oxyhemoglobin. FMRI sensitively and accurately captures and detects these changes, showing changes in brain function in real time. Resting-state functional magnetic resonance imaging (rsfMRI) primarily reflects the spontaneous activity of the brain at rest. RsfMRI has the characteristics of high temporal and spatial resolution and repeatability, the interference to the participants is relatively small, and no specific tasks need to be performed, which can shorten the inspection time and improve the compliance of patients, especially suitable for the development of clinical research (22, 23). Previous studies on the brain function of healthy people have found that rsfMRI reflects the differential changes in healthy groups under different emotional and environmental states (24), and can also reflect stable brain function differences between people (25–27). Some studies have examined sex differences in MDD using the rsfMRI method. Mei et al. (28) used ALFF to show that the bilateral caudate nucleus and posterior cingulate gyrus were the primary regions in the brain that differed between the sexes. In the above brain regions, males in MDD have lower ALFF than females in MDD and female HCs, and female in MDD have higher ALFF compared with male HCs. Yao et al. (29) found a wide range of ALFF values differences in brain regions between male in MDD and female in MDD, including in the frontoparietal, auditory, attention and cerebellum networks. These studies suggest brain functional differences between MDD patients of different sexes, and these differences provide neuroimaging evidence for exploring the sex-specific incidence and clinical manifestations of MDD. Due to the influence of sample size and inclusion criteria, the results of each study are different, suggesting that further research on this topic is required. It is of interest to further explore the sex differences in patients with MDD in the method of ALFF. To our knowledge, the influence of sex on brain function has not yet been fully explored using the regional homogeneity (ReHo) method in patients with MDD and studies combining ReHo and ALFF have not been found. In this study we utilized a whole-brain analysis of regional homogeneity and amplitude of low frequency fluctuation to examine sex differences in medication-naïve participants with MDD. ALFF is a commonly used analytical method in resting f-MRI. By calculating amplitudes with frequencies of 0.01–0.080 Hz, the strength of brain activity in this region is reflected. ReHo is a voxel-based method that estimates brain activity for a given voxel and its nearest neighbors by measuring the similarity or synchrony between time series using the Kendall coefficient (30). ReHo also has been widely used to study brain function in MDD. Sun et al. (31) used the ReHo method and found that patients with early-onset MDD and late-onset MDD have abnormal brain function in the default network. Yang et al. (32) studied 27 MDD patients with suicidal ideation (SI) and 24 MDD patients without SI using a whole-brain analysis of ReHo. They found that elevated cingulate activity may be associated with SI.

In this study, we utilized a whole-brain analysis of regional homogeneity and amplitude of low frequency fluctuation to examine sex differences in medication-naïve participants with MDD. Further understanding of the neurophysiological mechanism of brain function in MDD will provide the basis for the diagnosis and treatment of the disease in the future.

## Methods

### Participants

All patients were recruited from the outpatient department of the Psychiatry Department of the First Hospital of China Medical University. Healthy people were recruited *via* advertising. All enrolled patients were assessed for the diagnosis of MDD by two trained psychiatrists according to the Structured Clinical Interview for DSM-IV (SCID-I). A total of 77 MDD patients (30 men and 47 women) aged 18–51 years were enrolled. All patients met the following inclusion criteria: (1) the diagnostic criteria of MDD in SCID-1. (2) Hamilton Depression Scale (HAMD-17) score ≥17. (3) Without any other Axis I and Axis II disorders.

A total of 85 healthy people (31 men and 47 women) matched with the patient group in terms of gender, years of education and age were enrolled. All healthy controls met the following criteria: HAMD-17 score <7 points, no history of mental illness, and no family history of mental illness, without any other Axis I and Axis II disorders.

All enrolled people were excluded in the following situations:

(1) History of a Loss of Consciousness due to Head Trauma for 5 min or More or a History of Other Cerebral Organic Diseases. (2) Chronic Diseases, Such as Hypertension and Diabetes, That may Lead to Changes in Brain. (3) Endocrine Diseases That can Lead to Affective Disorders. (4) History of Substance Abuse and Alcohol Addiction. (5) A Contraindication to MRI Scans. (6) The Inability to Complete MRI Scans. (7) Pregnancy.

All enrolled participants had a detailed understanding of the experimental prohibited content, steps, purpose and related contraindications before enrolment. Written informed consent was signed after fully informed of the study to all participants. The study was approved by the Ethics Committee of China Medical University.

### Magnetic resonance imaging scan and processing

Imaging data were acquired by the GE Signa HDx 3.0 T MRI scanner at the First Hospital of China Medical University, Shenyang, China. The patient was instructed to rest with their eyes closed and to avoid thinking. Structure images were obtained by 3D-T1 three-dimensional rapid scrambled gradient echo sequence (3D-FSPGR) with the following scan parameters: repetition time (TR) = 7.1 ms, echo time (TE) = 3.15 ms, flip angle (FA) = 13°, number of slices = 176, slice thickness = 1 mm, voxel resolution = 1 × 1 × 1 mm^3^, image matrix = 240 × 240, field of view (FOV) = 240 × 240 mm, scan time:8 min 22 s. Spin echo planar imaging (EPI) was used to collect rsfMRI imagine under the following acquisition parameters: TR = 2,000 ms, echo time = 40 ms, matrix = 64 × 64; field of view = 240 × 240 mm^2^; slice thickness = 3 mm; slice interval = 0; slice number = 35 slices; scan time: 6 min 40 s.

The SPM12 software package and the DPABI software package were used to preprocess the data. Considering the stability of the machine and the adaptation of the enrolled population to the environment, the first 10 time points were removed. To correct for the time difference between layers and head movement, the head rotation did not exceed 3°, and the translation did not exceed 3 mm. Frame-wise displacement (FD) were calculated and no statistical difference were found in mean FD with the four groups, then regression of covariates, including brain white matter signal, cerebrospinal fluid signal and head movement parameter. The images underwent spatial normalization in the Montreal Neurological Institute space using DARTEL with a resampling voxel volume of 3 × 3 × 3 mm.

ReHo analyze: the linear detrend method was used to remove the influence of thermal noise, using a temporal bandpass filter retain the BOLD signal in the 0.01–0.08 Hz frequency band. The consistency between a voxel and the surrounding 26 adjacent voxels was calculated according to the Kendall coefficient of concordance (KCC). The KCC value of each voxel in the entire brain was divided by the mean value of the KCC of the voxels of the entire brain to obtain the normalized ReHo diagram. This process was followed by spatial smoothing with a 6 mm full-width at half-maximum Gaussian kernel smoothing of 6 × 6 × 6 mm.

ALFF analyze: Smoothing with a 6-mm full-width at half-maximum Gaussian kernel, linear detrend method, and then a fast Fourier transform (FFT) was performed to obtain the power spectrum. Obtain the mean square root of the ALFF measurement for each voxel in the range of 0.01–0.08 Hz. Finally, physiological high-frequency noise was eliminated using the temporal bandpass (0.01–0.08 Hz) filter.

### Statistical analyses

Subjective characteristics were conducted by SPSS 25.0 software (SPSS IBM Corporation, USA). Age, years of education, and total HAMD-17 scores among the four groups were analyzed by one-way analysis of variance (ANOVA). Two-way analysis of variance (ANOVA) between diagnosis (MDD and HC) and gender (male and female) has also been used to compare demographic data (age and education) and HAMD. Correlational analyses were performed to compare the correlation among the ALFF and ReHo values of the dysfunctions in the brain regions with the HAMA-17 and YMRS scores in females with MDD females and males with MDD groups. Independent sample *t* tests were utilized to compare the duration of illness between MDD women and MDD men. The statistical significance was determined by *p* < 0.05.

Correlational analyses were performed to find possible clinical correlation, by compare the correlation among the mean ALFF and ReHo values of the significant different brain regions with the total HAMA-17scores, scores of five clinical factors (cognitive disturbance; anxiety/somatization; psychomotor retardation; sleep disturbance; weight loss) in the HAMD and total HAMA scores in females and males with MDD groups.

The imaging data of brain regions in the four groups were subjected to a two-way analysis of variance (ANCOVA) between diagnosis (MDD and HC) and gender (male and female) as the main effects using SPM12. Statistical significance was determined by voxel *p* < 0.005 and cluster *p* < 0.05 [Gaussian random field (GRF) correction]. Significant diagnosis gender interactions were interpreted by performing a *post hoc* test separately for males and females in the HC and MDD groups (Bonferroni correction, *P* < 0.05).

## Results

### Demographic and clinical characteristics of participants

The demographics and clinical features of each group are listed in Table 1. Age, sex, or education years did not significantly differ among men with MDD, women with MDD, HC males, and HC females. The two patient subgroups showed significantly higher HAMD-17 scores than HCs, and no significant differences were found between men and women with MDD. There was no significant effect of diagnosis, sex or interaction of diagnosis and sex in age and education, the effect of diagnosis in HAMD-17 was significant, the duration of illness and disease state have no significantly different between the two MDD groups. The HAMD-17 scores were divided into five clinical factors and are listed in Table 2. No significant associations between ALFF/ReHo values and total HAMA-17 scores, clinical factors in the HAMD and total HAMA scores were found in female and male patients with MDD in correlation analyses.

**Table 1:** Demographics and clinical data of participants.

**Items**	**Healthy controls**		**MDD participants**		**t/F**	***p***
	**Male**	**Female**	**Male**	**Female**		
Numbers	31	47	30	47		
Age (years, mean ± S.D.)	27.94 ± 7.72	28.96 ± 10.18	29.53 ± 9.25	29.98 ± 10.18	0.307	0.821
Education (year, mean ± S.D.)	12.77 ± 3.14	13.17 ± 2.84	13.43 ± 3.04	13.00 ± 3.27	0.259	0.855
HAMD-17 (mean ± S.D.)	0.81 ± 1.42	1.36 ± 1.61			−1.56	0.072
HAMD-17 (mean ± S.D.)			26.50 ± 5.54	26.34 ± 6.04	0.117	0.907
HAMD-17 (mean ± S.D.)	0.81 ± 1.42	1.36 ± 1.61	26.50 ± 5.54	26.34 ± 6.04	453.40	0.000^*^
HAMA (mean ± S.D.)	0.52 ± 1.18	1.00 ± 1.56			−1.464	0.153
HAMA (mean ± S.D.)			21.00 ± 9.13	22.49 ± 8.86	−0.675	0.502
HAMA (mean ± S.D.)^a^	0.52 ± 1.18	1.00 ± 1.56	21.00 ± 9.13	22.49 ± 8.86	139.61	0.000^*^
Duration of illness (month, mean ± S.D.)^b^	N/A	N/A	11.14 ± 16.16	9.18 ± 9.67	0.39	0.535

MDD, major depressive disorder; HAMD-17, 17-item Hamilton Rating Scale for Depression;

* Significance level at *p* < 0.05; N/A, not applicable.

a Missing information for 2 MDD female participants,4 MDD male participant and 1 healthy control.

b Missing information for 5 MDD female participants and 4 MDD male participants.

**Table 2:** Data of the five clinical factors in HAMD of male and female MDD.

**Items**	**MDD male**	**MDD female**	***P***
Cognitive disturbance	4.73 ± 2.35	4.45 ± 1.97	0.811
Anxiety/somatization	6.43 ± 2.30	6.74 ± 2.92	0.552
Psychomotor retardation	8.33 ± 2.60	8.66 ± 2.15	0.623
Sleep disturbance	4.37 ± 1.50	4.30 ± 1.73	0.940
Weight	1.13 ± 0.94	0.70 ± 0.86	0.046^*^

Cognitive disturbance: HAMD 2, 3, 9; anxiety/somatization: HAMD 10, 11, 12, 15, 17; psychomotor retardation: HAMD 1, 7, 8, 14; sleep disturbance: HAMD 4, 5, 6; weight: HAMD 16.

* Significance level at *p* < 0.05.

### ALFF and ReHo alterations among four groups

**Figure 1:**
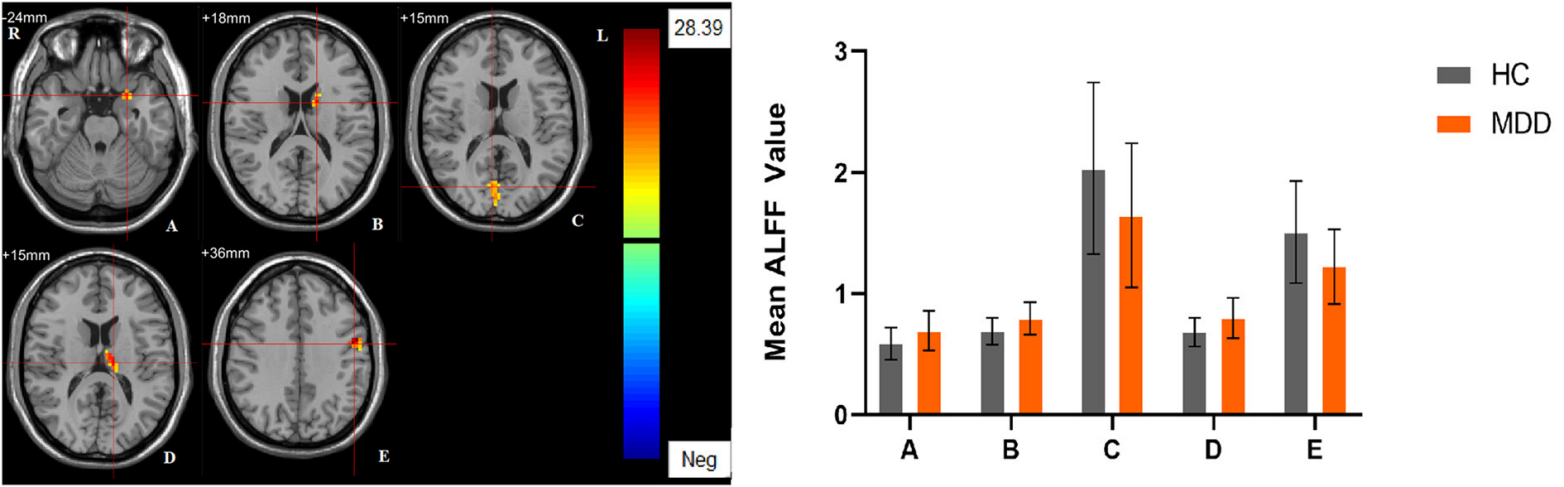
The main effect of diagnostic group. Regions with a main effect of the diagnostic group included **(A)** The left Olfactory and the left orbital inferior frontal gyrus; **(B)** The left caudate; **(C)** The bilateral calcarine; **(D)** The left thalamus; **(E)** The left precentral gyrus and the left postcentral gyrus (cluster-level threshold of *p* < 0.05 after GRF correction). The color bar represents the range of F values. R = right, L = left. The graph shows the ALFF values (mean ± standard deviation) extracted from regions with a main effect of diagnostic group. The Y-axis represents ALFF values. The X-axis represents regions with a main effect of the diagnostic group.

**Figure 2:**
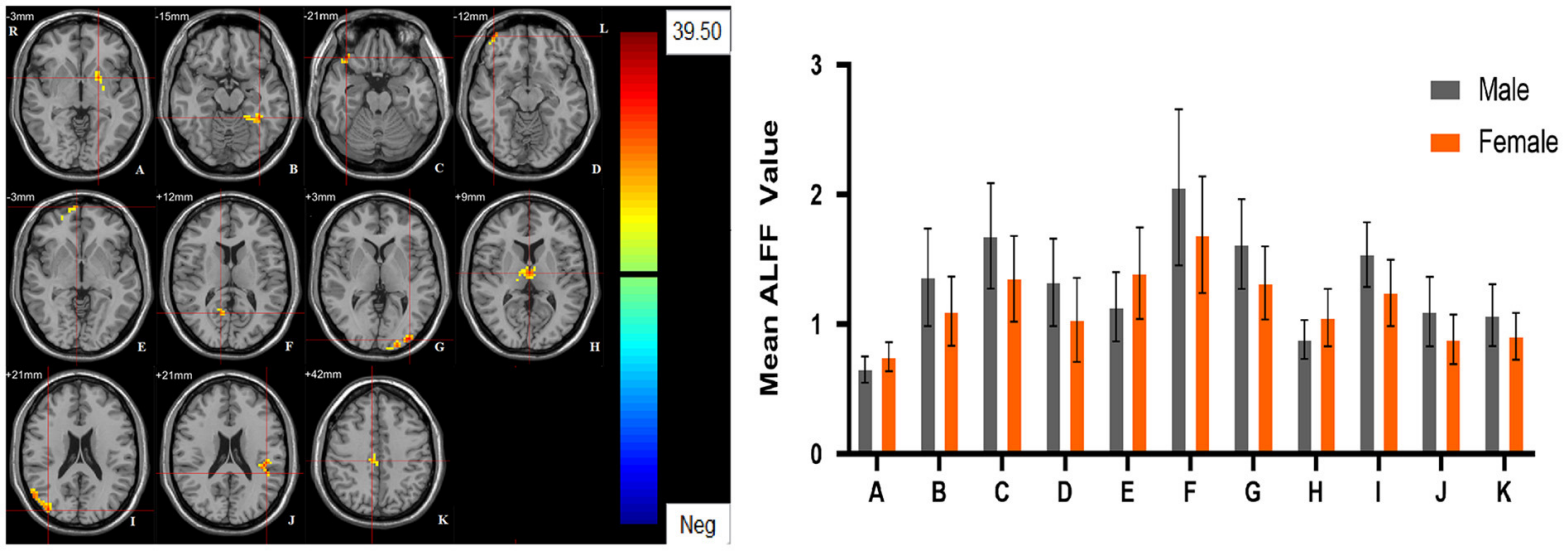
The main effect of sex group. Regions with a main effect of sex group included **(A)** The left putamen; **(B)** The left lingual gyrus, the left calcarine and the left inferior temporal gyrus; **(C)** The right orbital inferior frontal gyrus; **(D)** The right orbital middle frontal gyrus; **(E)** The right orbital medial frontal gyrus; **(F)** The right calcarine; **(G)** The left middle occipital lobe; **(H)** The bilateral thalamus; **(I)** The right middle occipital lobe; **(J)** The left superior temporal gyrus; **(K)** The right median cingulate gyrus (cluster-level threshold of *p* < 0.05 after GRF correction). The color bar represents the range of F values. R = right, L = left. The graph shows the ALFF values (mean ± standard deviation) extracted from regions with a main effect of sex group. The Y-axis represents ALFF values. The X-axis represents regions with a main effect of sex group.

**Figure 3:**
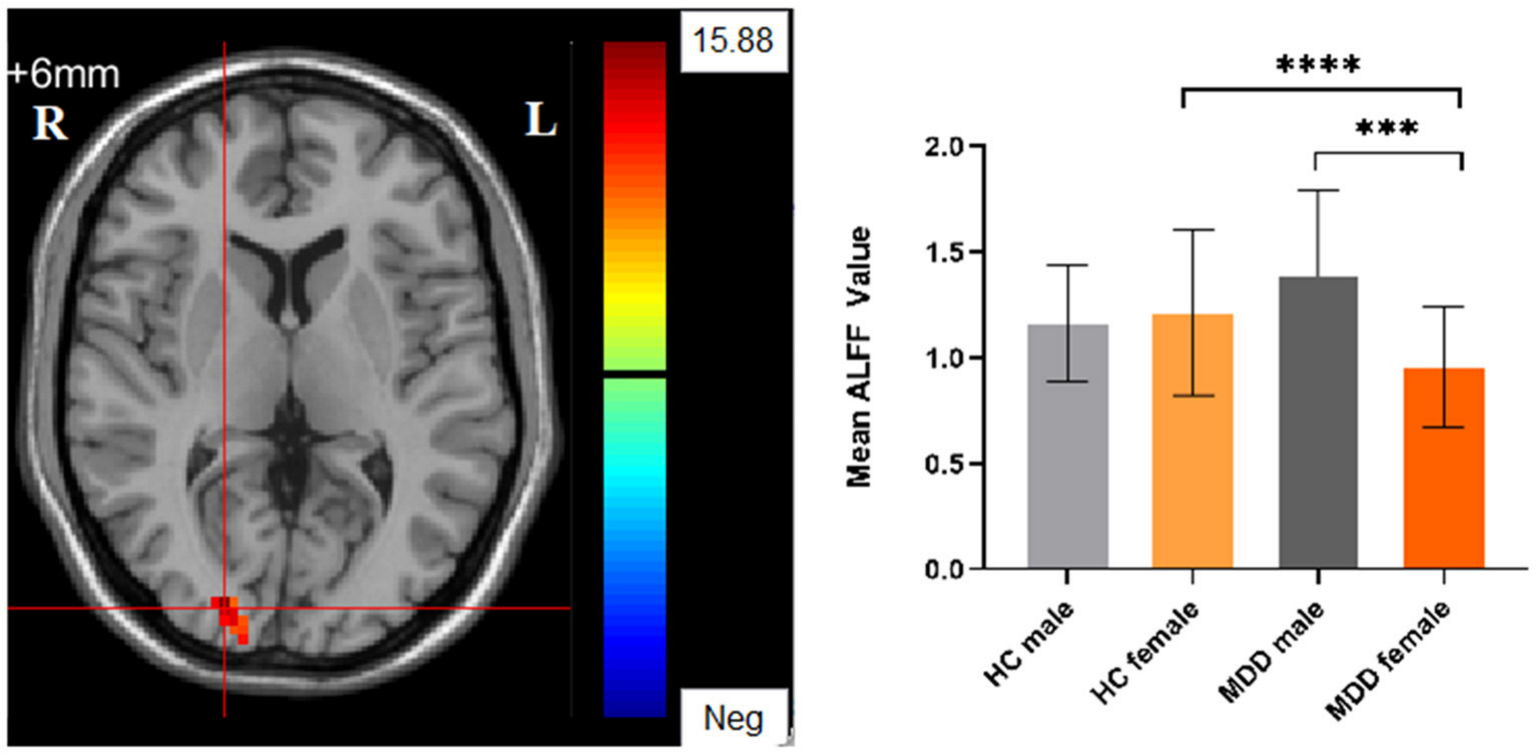
The diagnosis group × gender group interaction. Regions with diagnosis group × sex group interactions include the right superior occipital gyrus (cluster-level threshold of *p* < 0.05 after GRF correction). The color bar represents the range of F values. R = right, L = left. The graph shows the ALFF values (mean ± standard deviation) extracted from regions with the diagnosis groups × sex group interaction. The Y-axis represents ALFF values. The X-axis represents 4 groups of participants. ****p < 0.001; ***p < 0.005.

Significant different ALFF values in the main effect of diagnosis were observed in several brain regions, including bilateral calcarine, left caudate, left postcentral gyrus, left precentral gyrus, left thalamus and left orbital inferior frontal gyrus. The ALFF values in patients with MDD was significantly increased in the left caudate, left thalamus, left orbital inferior frontal gyrus, and significantly decreased in the bilateral calcarine, left postcentral gyrus, and left precentral gyrus compared with HCs (Table 3, Figure 1). Significant different ALFF values were also observed between the sexes in several brain regions, including the left putamen, left lingual gyrus, left inferior temporal gyrus, bilateral calcarine, bilateral thalamus, bilateral middle occipital gyrus, left superior temporal gyrus, right median cingulate gyrus, right orbital inferior frontal gyrus, right orbital middle frontal gyrus, right orbital medial gyrus. The female participants exhibited significantly increased ALFF in the left putamen, right orbital medial gyrus, and bilateral thalamus and significantly decreased ALFF in the left lingual gyrus, left inferior temporal gyrus, bilateral calcarine, bilateral middle occipital gyrus, left superior temporal gyrus, right median cingulate gyrus, right orbital inferior frontal gyrus, right orbital middle frontal gyrus compared to male participants (Table 3, Figure 2). The right superior occipital gyrus was significantly observed in diagnosis sex interaction. A *post hoc* test indicated that MDD females had reduced ALFF in right superior occipital gyrus compared with HC females (*P* < 0.001, Bonferroni correction) and MDD males (*P* < 0.005, Bonferroni correction) (Table 3, Figure 3).

**Table 3:** Clusters exhibiting the influence of male and female sex on ALFF in the MDD and HC groups.

**Brain area**	**Cluster size**	**Peak MNI coordinates**			**Peak F value**
		**X**	**Y**	**Z**	
Main effect of diagnosis groups					
A The left Olfactory The left orbital inferior frontal gyrus	44	−24	9	−24	20.81
B The left caudate	73	−15	3	18	22.61
C The right calcarine The left calcarine	98	6	−75	15	14.03
D The left thalamus	31	−12	−18	15	21.65
E The left precentral gyrus The left postcentral gyrus	60	−51	−3	36	28.39
Main effect of gender groups					
A The left putamen	77	−21	6	−3	17.32
B The left lingual gyrus The left calcarine The left inferior temporal gyrus	114	−36	−42	−15	22.17
C The right orbital inferior frontal gyrus	57	39	30	−21	39.5
D The right orbital middle frontal gyrus	48	42	57	−12	24.27
E The right orbital medial frontal gyrus	36	6	69	−3	15.16
F The right calcarine	28	12	−60	12	16.44
G The left middle occipital lobe	184	−36	−93	3	29.42
H The bilateral thalamus	40	0	−12	9	23.65
I The right middle occipital lobe	139	39	−84	21	22.32
J The left superior temporal gyrus	69	−45	−39	21	24.82
K The right median cingulate gyrus	31	9	−24	42	14.45
Diagnosis group×Gender groups interaction					
The right superior occipital gyrus	24	21	−90	6	15.88

These findings correspond to a corrected *P* < 0.05 by GRF correction.

**Figure 4:**
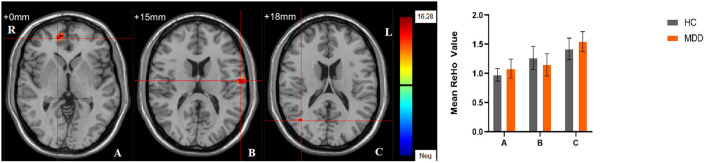
The main effect of diagnostic group. Regions with a main effect of the diagnostic group included **(A)** The right superior medial frontal gyrus; **(B)** The left postcentral gyrus; **(C)** The right middle temporal gyrus (cluster-level threshold of *p* < 0.05 after GRF correction). The color bar represents the range of F values. R = right, L = left. The graph shows the ReHo values (mean ± standard deviation) extracted from regions with a main effect of diagnostic group. The Y-axis represents ReHo values. The X-axis represents regions with a main effect of the diagnostic group.

**Figure 5:**
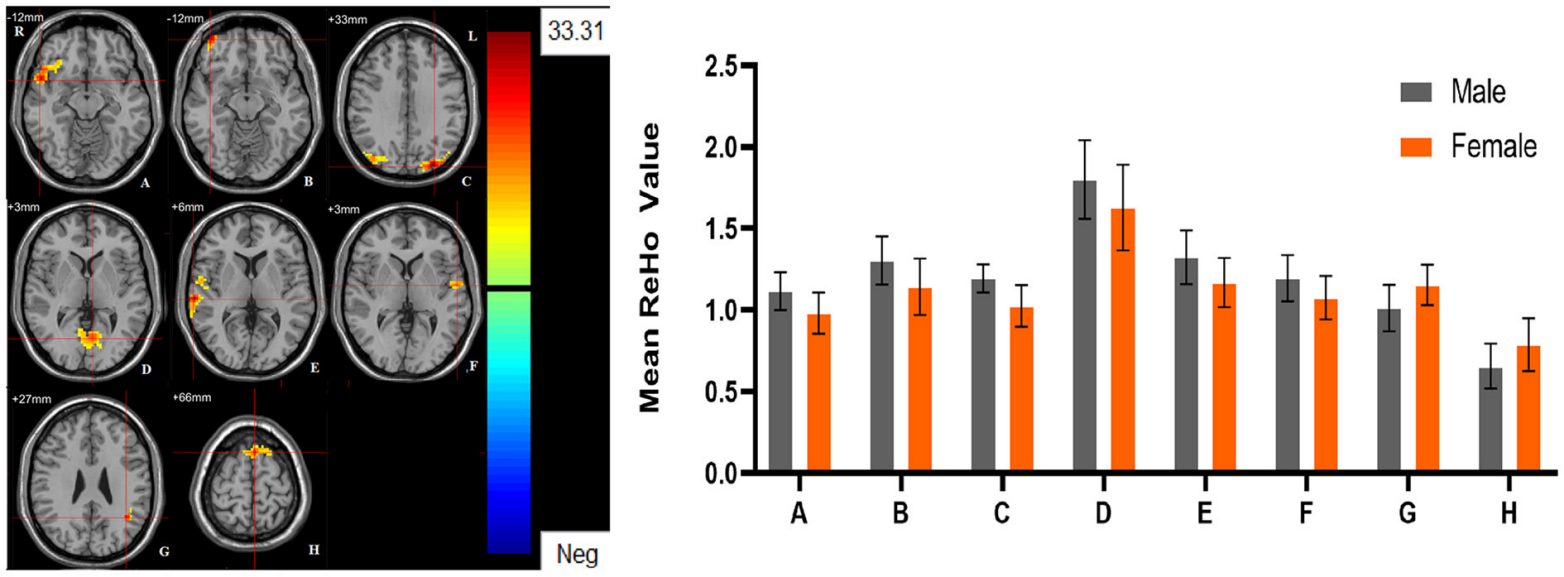
The main effect of sex group. Regions with a main effect of sex group included **(A)** The right orbital inferior frontal gyrus; **(B)** The right orbital middle frontal gyrus; **(C)** The left middle occipital lobe, and the right postcentral gyrus; **(D)** The bilateral lingual gyrus and the bilateral calcarine; **(E)** The right superior temporal gyrus; **(F)** The left superior temporal gyrus; **(G)** The left middle temporal gyrus; **(H)** The left dorsolateral Superior frontal gyrus (cluster-level threshold of *p* < 0.05 after GRF correction). The color bar represents the range of F values. R = right, L = left. The graph shows the ALFF values (mean ± standard deviation) extracted from regions with a main effect of sex group. The Y-axis represents ALFF values. The X-axis represents regions with a main effect of sex group.

**Figure 6:**
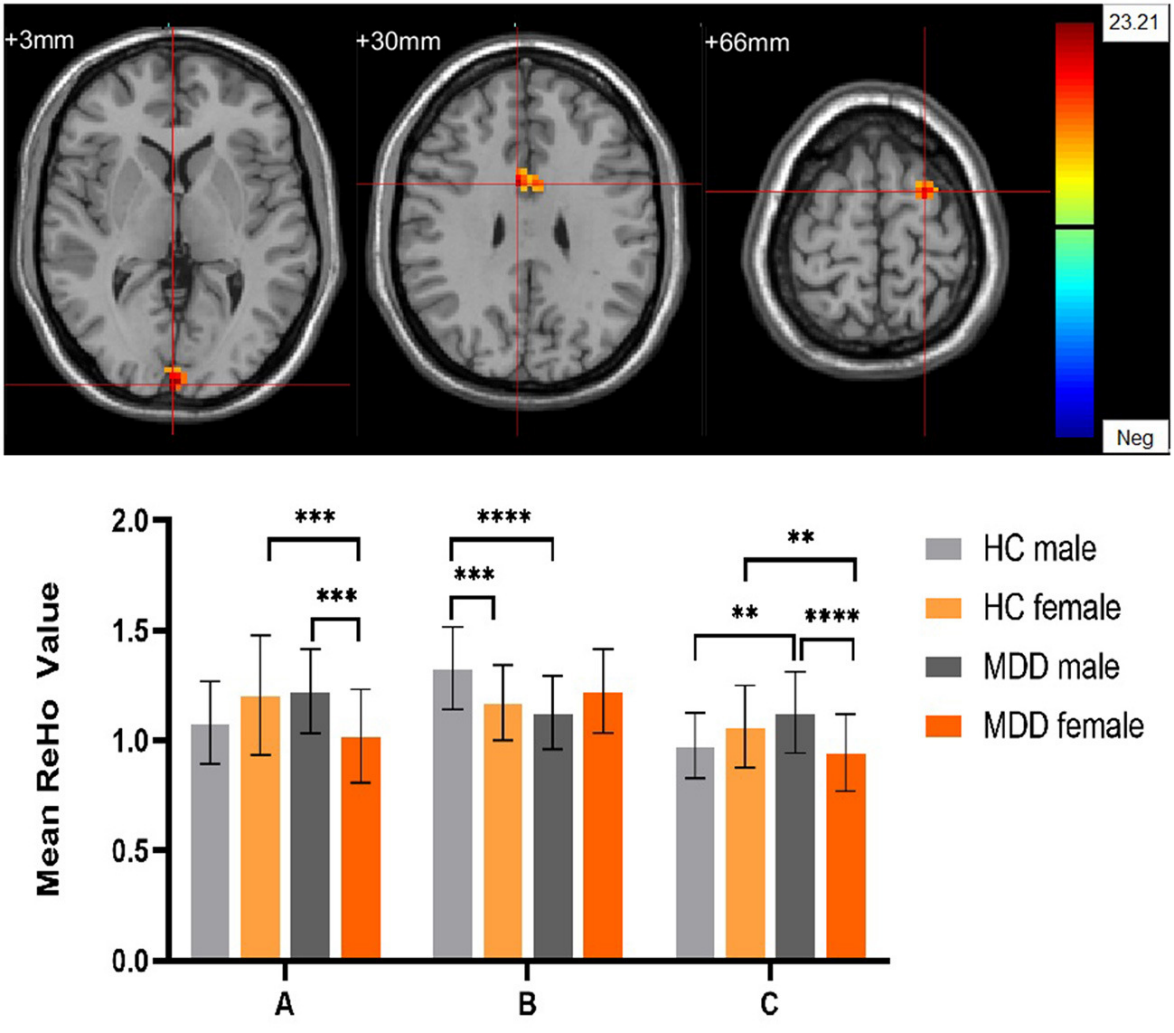
The diagnosis group × gender group interaction. Regions with diagnosis group × sex group interactions include **(A)** The left Calcarine; **(B)** The right median cingulate gyrus; **(C)** The left dorsolateral superior frontal gyrus (cluster-level threshold of *p* < 0.05 after GRF correction). The color bar represents the range of F values. R = right, L = left. The graph shows the ReHo values (mean ± standard deviation) extracted from regions with the diagnosis groups × sex group interaction. The Y-axis represents ReHo values. The X-axis represents regions with the diagnosis groups × gender group interaction. ****p < 0.001; ***p < 0.005; **p < 0.01.

Significant different ReHo values in the main effect of diagnosis were observed in the right superior medial frontal gyrus, left postcentral gyrus, right middle temporal gyrus. The ReHo values in patients with MDD was significantly increased in the right superior medial frontal gyrus, right middle temporal gyrus, and significantly decreased in the left postcentral gyrus, compared with HCs (Table 4, Figure 4). In main effect of sex, significant different ReHo value were observed in the bilateral superior temporal gyrus, bilateral lingual gyrus, bilateral calcarine, right orbital inferior frontal gyrus, right orbital middle frontal gyrus, right postcentral gyrus, left middle occipital gyrus, left dorsolateral superior frontal gyrus, left middle temporal gyrus. The female participants exhibited significantly increased ReHo in the left dorsolateral superior frontal gyrus, left middle temporal gyrus, and significantly decreased ReHo in the bilateral superior temporal gyrus, bilateral lingual gyrus, bilateral calcarine, right orbital inferior frontal gyrus, right orbital middle frontal gyrus, right postcentral gyrus, left middle occipital gyrus compared to male participants (Table 4, Figure 5). The left Calcarine, right median cingulate gyrus, left dorsolateral superior frontal gyrus were significantly observed in diagnosis sex interaction. A *post hoc* test indicated that women with MDD had significantly decreased ReHo value in the left Calcarine compared with HC females (*P* < 0.005, Bonferroni correction) and men with MDD (*P* < 0.005, Bonferroni correction). In the left dorsolateral superior frontal gyrus, women with MDD had significantly decreased ReHo value compared with HC females (*P* < 0.01, Bonferroni correction), while men with MDD had significantly increased ReHo compared with HC males (*P* < 0.01, Bonferroni correction) and women with MDD (*P* < 0.001, Bonferroni correction). Furthermore, the ReHo in the right median cingulate gyrus of HC males was higher than that in HC females (*P* < 0.005, Bonferroni correction), and men with MDD (*P* < 0.001, Bonferroni correction) (Table 4, Figure 6).

**Table 4:** Clusters exhibiting the influence of male and female sex on ReHo in the MDD and HC groups.

**Brain area**	**Cluster size**	**Peak MNI coordinates**			**Peak F value**
		**X**	**Y**	**Z**	
A The right superior medial frontal gyrus	41	15	51	0	16.28
B The left postcentral gyrus	64	−60	−9	15	15.34
C The right middle temporal gyrus	35	39	−66	18	13.15
Main effect of gender groups					
A The right orbital inferior frontal gyrus	218	54	9	−12	27.66
B The right orbital middle frontal gyrus	188	42	57	−12	30.02
C The left middle occipital lobe the right postcentral gyrus	1,654	−30	−87	33	33.31
D The bilateral lingual gyrus the bilateral calcarine	196	−6	−69	3	19.96
E The right superior temporal gyrus	186	66	−24	6	30.32
F The left superior temporal gyrus	77	−57	−6	3	16.71
G The left middle temporal gyrus	59	−39	−54	27	23.84
H The left dorsolateral Superior frontal gyrus	105	−3	18	66	23.41
Diagnosis group×Gender groups interaction					
A The left Calcarine	66	3	−99	3	23.21
B The right median cingulate gyrus	27	6	6	30	20.61
C The left dorsolateral superior frontal gyrus	40	−24	3	66	19.51

These findings correspond to a corrected *P* < 0.05 by GRF correction.

## Discussion

In this study, we observed sex-specific patterns and diagnostic differences in MDD Patients, our further *post hoc* comparisons indicated that women with MDD showed decreased ALFF value in the right superior occipital gyrus and decreased ReHo value in the left calcarine and left dorsolateral superior frontal gyrus compared with HC females and men with MDD. Men with MDD showed decreased ReHo value in the right median cingulate gyrus compared with HC males and increased ReHo value in the left dorsolateral superior frontal gyrus compared with HC males, we also found that in HC males showed increased ReHo value in the right median cingulate gyrus compared with HC females. The findings revealed that there were sex differences in brain functions in MDD patients and may related to the incidence and clinical features of MDD men and women, this may lead to sex-specific dysfunction in MDD.

The occipital lobe is mainly related to the processing of visual information, and damage to the occipital lobe may lead to a decline in individual cognitive tasks related to vision. Although visual impairment is not included as a core symptom of depressive disorders, studies (33, 34) have found that MDD patients without eye disease have decreased visual function compared with normal people. MRI high-resolution mapping data showed that MDD patients have reductions in myelin in the occipital cortex compared to healthy controls (35). Previous studies have also found abnormal brain structure and function changes in the occipital lobe of MDD patients (36, 37). Calcarine is a concentration of the primary visual cortex of the occipital lobe. Previous research (38) found that calcarine neurological abnormalities may lead to impairment of MDD cognitive control function by affecting visual processing and visual attention. Piani et al. (39) indicted that there was diagnosis by sex effects in the right calcarine cortex by the methods of GMV. The abnormalities found in women with MDD compared with HC women and MDD men in our study suggested that occipital lobe may be one of the neurological biomarkers in MDD women, and may lead to visual impairment and visual cognitive impairment in women with MDD.

The cingulate gyrus is a limbic structure that thought to contribute to motivational aspects of pain and related to responds selection. Previous studies suggested that cingulate gyrus has highly interconnected with amygdala, orbitofrontal cortex (OFC), insular and hippocampus (40). Social pain, error made and conflict between possible responds can active cingulate gyrus (41). Previous studies in MDD were focused more on anterior cingulate gyrus (ACG) and posterior cingulate gyrus (PCG), the median cingulate gyrus (DCG) is also an important limbic structure that participant in salient stimuli processing (42). Studies were found that disfunction of median cingulate gyrus is related to negative emotions (43). Su et al. (44) found that the abnormalities of GMW in bilateral DCG are associated with anorexia nervosa. A meta-analysis has found VBM abnormalities in the DCG in patients with behavior addictions (45). A previous study with a total of 11,246 adult participants found that the symptoms of poor appetite and addictive problem were significantly higher in males compared with MDD women (46), this result has also been confirmed in other studies (11). The decreased ReHo values found in the right DCG in MDD males compared with HC males in our study indicated that the abnormalities of DCG may help to support that MDD males may have more problems in poor appetite and addiction problems.

DLPFC is an important part of PFC that thought to be associated with “cognitive” or “executive” functions (47), receiving input from specific sensory cortices, and regulate emotion through projections to the medial PFC and cingulate cortices (48). Recent studies have found that the imbalance in DLPFC activity may contribute to depression (49, 50). According to the guidelines on the repetitive transcranial magnetic stimulation (rTMS), the level A evidence was reached for high-frequency (HF) rTMS of the left DLPFC for depression (51). Previous studies have found that there were sex differences in the expression of glutamatergic genes and transcription of monoamine in the DLPFC of MDD patients (52, 53). A recent study explored the effects of sex on the neural response to acute psychosocial stress of MDD, and they found that men exhibited higher DLPFC activity than women (54). The above studies supported our results that there are sex differences in DLPFC in MDD patients, with unclear potential neurological mechanism. suggesting that the DLPFC may be the biomarker brain regions that can help diagnose and treat MDD in both men and women, further studies of DLPFC are required.

We also found gender differences of cortical-limbic-thalamus-striatal neural system in ALFF and gender differences of cortical-limbic neural system in ReHo. The orbital frontal gyrus (OFC), temporal gyrus, and occipital gyrus were found to have significantly gender difference in both ALFF and ReHo in all participants.

OFC is an important part of limbic system and have tight connection with with ACC and PCC, Goldstein et al. (55) found that the OFC were activated in response to stress. The temporal lobe is involved in many different functions of the brain, such as gauging distance, recognizing faces, emotion recognition, reading comprehension, etc. A previous study showed that the temporal lobe is involved in the formation of anxiety (56). Kimbrell et al. (57) found that increased regional cerebral blood flow (rCBF) in the left temporal lobe and decreased regional cerebral blood flow in the right temporal lobe were associated with anxiety and anger, as assessed by PET. Sex abnormalities of ALFF also were observed in the left putamen and bilateral thalamus in all participants and HCs; Previous studies have found that patients with MDD show enhanced nodal connectivity in the putamen, which is positively correlated with the number of depressive episodes (58). The thalamus is an important sensory transmission area in the brain and a key relay station that transmits nociceptive messages to the cerebral cortex. Previous fMRI studies found that Painful stimulation can activate the thalamus (59). Sex abnormalities of ReHo were also observed in the postcentral gyrus, which is the sensory center of the body (60), and responds to sensory stimuli, such as touch, pressure, temperature and pain (61). The postcentral gyrus is an important part of the pain perception circuit in the brain, and abnormalities in the central posterior gyrus may lead to the development of chronic pain (62). These above different brain regions found in our study suggest us that there are sex-specific differences in males compared with females, which may influence the incidence and clinical features of MDD men and women.

This study has several limitations. First, this work was limited to a cross-sectional study, where the effects of drugs and disease progression cannot be observed on patients. Future longitudinal studies need to be conducted to provide further guidance for different changes in brain function between men and women with depression. Second, due to the limited number of participants, patients with MDD did not undergo a more detailed subtype classification, future studies will increase sample sizes, different subtypes, conduct a more detailed analysis of differences in brain function in men and women with depression. Finally, our enrolled patients have a large age span of onset, which may have an impact on brain function and structure, and more detailed studies can be conducted to further investigate the effect of MDD onset age on brain function and structure.

## Conclusion

In summary, we used the methods of ALFF and ReHo to explore the differences in brain activity between male and female. We found that men and women do have sex differences in brain function, the occipital lobe, calcarine, DLPFC, and DCG were the main different brain regions found in our study between male and female in MDD, which may be the biomarker brain regions that can help diagnose and treat MDD in men and women, these findings revealed that there were sex differences in brain functions in MDD patients and further detailed investigation of sex differences in MDD in the future can make sense.

## Data availability statement

The raw data supporting the conclusions of this article will be made available by the corresponding author.

## Ethics statement

The studies involving human participants were reviewed and approved by the Medical Scientific Research Ethics Committee of the First Hospital of China Medical University. The patients/participants provided their written informed consent to participate in this study.

## Author contributions

YT, LK, FW, and ZT designed the study. LK, FW, and ZT recruited the patients. ZT and XJ acquired the data. ZT and LK analyzed the data. ZT wrote the article. YT was responsible for project administration and funding acquisition. All authors contributed to the article and approved the submitted version.

## Funding

This work was supported by grants from the National Key R&D Program of China (Grant Nos. 2018YFC1311600 and 2016YFC1306900 to YT), Liaoning Revitalization Talents Program (Grant No. XLYC1808036 to YT), Natural Science Foundation of Liaoning Province (2020-MS-176 to XJ), National Key R&D Program Science and Technology Winter Olympics (2021YFF0306503 to FW), Joint Fund of National Natural Science Foundation of China (U1808204 to FW), and Natural Science Foundation of Liaoning Province (2019-MS-05 to FW).

## Conflict of interest

The authors declare that the research was conducted in the absence of any commercial or financial relationships that could be construed as a potential conflict of interest.

## Publisher's note

All claims expressed in this article are solely those of the authors and do not necessarily represent those of their affiliated organizations, or those of the publisher, the editors and the reviewers. Any product that may be evaluated in this article, or claim that may be made by its manufacturer, is not guaranteed or endorsed by the publisher.
